# Effects of dual-wavelength laser combined with periodontal flap surgery on periodontal parameters and inflammatory factors in patients with periodontic-endodontic lesions

**DOI:** 10.3389/fcimb.2026.1773155

**Published:** 2026-03-16

**Authors:** Lijie Xu, Yanjie Chai, Shenxi Yao, Meijie Shen

**Affiliations:** Department of Stomatology, Tongxiang First People’s Hospital, Tongxiang, Zhejiang, China

**Keywords:** dual-wavelength laser, inflammatory factors, periodontal flap surgery, periodontal parameters, periodontic-endodontic lesions

## Abstract

**Objective:**

This study investigated the clinical effectiveness of dual-wavelength laser with periodontal flap surgery in managing periodontic-endodontic lesions (PEL) and its impact on periodontal parameters and inflammatory markers.

**Methods:**

A total of 106 PEL patients were randomly assigned to a study group or a control group (53 each). All patients first received root canal treatment. The control group underwent conventional periodontal flap surgery, whereas the study group received adjunctive dual-wavelength laser treatment (Nd: YAG for root canal disinfection and Er: YAG for periodontal pocket debridement). Pain visual analogue scale (VAS) scores were recorded prior to treatment and at 1 and 7 days postoperatively. Periodontal parameters, gingival crevicular fluid (GCF) and serum inflammatory markers, and bacterial infection/clearance rates were assessed at baseline and 3 months post-treatment. Clinical efficacy and incidences of tooth mobility, periodontitis recurrence, and percussion pain were evaluated at 6 months.

**Results:**

Ninety-nine patients completed follow-up (48 in the study group and 51 in the control group). On postoperative day 1, VAS scores increased in both groups but were significantly lower in the study group (*P* < 0.05); by day 7, VAS scores declined with no between-group difference (*P* > 0.05). At 3 months, PD, CAL, GI, PLI, and levels of IL-1β, IL-6, and MMP-8 decreased in both groups, with greater improvements in the study group (*P* < 0.05). The study group also exhibited lower bacterial infection rates and higher bacterial clearance (*P* < 0.05). At 6 months, the study group showed higher treatment success and lower rates of tooth mobility, recurrence, and percussion pain (*P* < 0.05).

**Conclusion:**

Dual-wavelength laser adjunctive therapy enhances the clinical efficacy of periodontal flap surgery in PEL, significantly improving periodontal healing, reducing inflammation, and promoting bacterial clearance.

## Introduction

Periodontitis is a chronic inflammatory disease affecting the tooth‐supporting tissues and is primarily initiated by pathogenic microorganisms (Li et al., 2021). Among the various periodontal conditions, combined periodontic–endodontic lesions (PEL) represent a particularly challenging entity due to the simultaneous involvement of both pulpal and periodontal tissues. In PEL, bacterial toxins and inflammatory mediators may disseminate through lateral canals, accessory canals, or dentinal tubules, thereby inducing periodontal ligament destruction and alveolar bone resorption (Fang et al., 2021). Autoimmune reactions and inflammatory cascades further exacerbate disease progression, while the intimate anatomical connection between pulp and periodontium enables bidirectional infection spread, resulting in complex lesion patterns (Guo et al., 2022). Clinically, PEL often presents with mixed bacterial infections, variable symptoms, and overlapping radiographic features, making diagnosis, treatment planning, and prognosis assessment particularly difficult (Khojaste et al., 2022).

Patients with PEL typically experience a prolonged disease course and recurrent symptoms, substantially affecting oral function and quality of life. Current treatment strategies for PEL mainly rely on basic periodontal therapy, regenerative procedures, and surgical interventions (Dong et al., 2023). Periodontal flap surgery remains an essential approach for eliminating deep periodontal pockets and improving access for debridement (Ari et al., 2023). This technique is designed to minimize tissue trauma (Silviya et al., 2022), stabilizes the flap, and preserves interdental papilla integrity, thereby improving wound closure and periodontal healing. However, complete bacterial eradication is often difficult to achieve with surgery alone, particularly in anatomically complex sites.

Laser-assisted periodontal therapy has gained increasing attention in recent years due to its unique advantages. Laser irradiation is highly effective in removing subgingival calculus, eliminating pathogenic bacteria, and improving hemostasis and visibility during surgery (Jiang et al., 2022). Lasers can also modulate inflammation, promote coagulation, and accelerate early wound healing. As an adjunct to surgery, laser use may enhance connective tissue attachment, reduce epithelial down-growth, and support periodontal regeneration (Saha et al., 2023). Dual-wavelength laser systems may further provide complementary advantages. Nd: YAG laser–assisted therapy effectively reduces deep periodontal infection and promotes soft-tissue healing (Dortaj et al., 2022), whereas Er: YAG laser offers precise ablation of root surfaces and subgingival deposits with minimal thermal damage, facilitating effective decontamination and improved healing (Aoki et al., 2024). Moreover, a recent randomized controlled trial combining Er: YAG + Nd: YAG laser with minimally invasive surgery for intrabony defects indicates that dual-wavelength laser–assisted surgery is a feasible and potentially beneficial option (Skurska et al., 2025).

Early and appropriate periodontal intervention plays a crucial role in facilitating the healing of pulpal and periapical tissues (Yan et al., 2021). However, previous studies have primarily focused on root canal therapy or traditional periodontal procedures, and evidence regarding the combined application of dual-wavelength lasers with flap surgery for PEL remains limited. Therefore, this work aimed to elucidate the clinical efficacy of dual-wavelength laser therapy combined with periodontal flap surgery in patients with PEL, with particular emphasis on its effects on periodontal parameters and inflammatory mediators. The findings may offer new insights into optimizing therapeutic strategies and improving long-term periodontal and pulpal tissue healing.

## Materials and methods

### Ethical approval

The study was approved by our hospital’s Ethics Committee of Tongxiang First People’s Hospital, and all participants provided written informed consent.

### Sample size calculation

The sample size was calculated using the change in the gingival index (GI) at 3 months postoperatively as the primary outcome. According to pilot data, the expected GI values were 0.38 ± 0.27 in the study group and 0.57 ± 0.28 in the control group, yielding a mean difference δ = 0.19 and a pooled standard deviation σ = 0.275. With a two-sided significance of α = 0.05 and a power of 1-β = 0.90, and assuming an equal allocation ratio (n1 = n2), the required sample size was calculated using PASS 2020 software according to the formula: n1 = n2 = [2(Z_α_/_2_+Z_β_)^2^(σ^2^)]/(δ)^2^. The computation indicated approximately 46 participants per group. Considering that clinical trials may experience up to 20% attrition, and applying a conservative 15% anticipated dropout rate, the final sample size was set at 53 participants per group, totaling 106 participants.

### General information

From May 2024 to April 2025, 106 patients with PEL treated at Tongxiang First People’s Hospital were enrolled and randomly assigned to the study or control group (53 each). The study group comprised 31 males and 22 females aged 23–65 years (mean 40.26 ± 8.58), with disease duration of 3–28 months (mean 12.58 ± 5.19). Lesion types included 22 periodontic-origin cases, 18 endodontic-origin cases, and 13 true combined lesions. Lesion sites involved 11 anterior teeth, 19 premolars, and 23 molars; 24 participants reported a smoking history. The control group consisted of 27 males and 26 females aged 25–63 years (mean 42.30 ± 8.05), with disease duration of 4–26 months (mean 12.00 ± 5.27). Lesion types were 19 periodontic-origin, 19 endodontic-origin, and 15 true combined lesions; lesion locations included 8 anterior teeth, 18 premolars, and 27 molars. Nineteen participants were smokers. There were no differences between the groups in sex, age, disease duration, lesion type, lesion location, or smoking history (*P* > 0.05), indicating good baseline comparability.

### Randomization method and blinding method

Grouping was carried out using the random number table method: A total of 106 patients were numbered from 1 to 106 according to the order of enrollment. The corresponding number of random numbers was extracted from the “Random Number Table”. After arranging the values in ascending order, the first 53 cases were assigned to the control group and the last 53 cases to the study group. The grouping plan was prepared and sealed by an independent statistician and was unsealed by the researchers before the implementation of the intervention. Due to the nature of the intervention measures, it was not possible to blind the personnel implementing the intervention plan in this study. However, to reduce bias, the data testers and evaluators, as well as the data analysts, were blinded in this study. All evaluation materials were only labeled with patient numbers. Subjective indicators were independently filled out by patients and then sealed and submitted. The evaluators only processed anonymous data.

### Inclusion criteria

Patients were eligible for inclusion if they met all of the following conditions: (1) A confirmed diagnosis of combined PEL based on clinical examination, radiographs, and pulp vitality testing, with evident periodontal pockets and pulp involvement, in accordance with the diagnostic criteria established by the Chinese dental expert group (Chen et al., 2024); (2) Aged 18–65 years, with stable systemic health and the ability to comply with treatment and follow-up requirements; (3) The affected tooth was a permanent tooth with mobility ≤ grade II and root resorption ≤ one-third of root length, and judged to be retainable; (4) No use of antibiotics, nonsteroidal anti-inflammatory drugs, or prior periodontal therapy within 3 months before treatment; (5) Ethical approval obtained, and all patients provided written informed consent.

### Exclusion criteria

Patients were excluded if any of the following conditions applied: (1) Failure to meet diagnostic criteria for PEL, or presence of isolated periodontitis or isolated pulp disease; (2) Systemic diseases that significantly impair periodontal health (e.g., uncontrolled diabetes, osteoporosis, immune deficiency) or severe systemic comorbidities that contraindicate surgery (e.g., heart disease, hepatic or renal failure, hematologic disorders); (3) Pregnancy or breastfeeding, due to insufficient safety evidence regarding laser therapy; (4) Affected tooth being diagnosed as vertical root fracture through preoperative clinical examination, periapical radiograph screening, and intraoperative direct-vision exploration, or having severe tooth defects that could not be repaired and calcified root canals; (5) Furcation involvement of grade III or IV; (6) Known allergies to materials used in laser therapy, local anesthetics, or antibiotics; (7) History of psychiatric disorders, cognitive impairment, or communication barriers preventing adherence to treatment or follow-up; (8) Concurrent participation in other clinical trials that may interfere with study outcomes; (9) Long-term use of immunosuppressants or glucocorticoids, or a history of radiotherapy that may impair periodontal healing.

### Exclusion and withdrawal criteria

Participants were removed from the study under any of the following circumstances: (1) Discovery during the study that the patient did not meet inclusion criteria or met undisclosed exclusion criteria, indicating erroneous enrollment; (2) Failure to complete the assigned treatment protocol (e.g., withdrawal, treatment change, transfer to another institution, or loss to follow-up); (3) Occurrence of severe adverse events during treatment or follow-up that rendered continuation unsafe or inappropriate; (4) Non-adherence to medical instructions, such as self-administration of antibiotics or analgesics, or receiving additional periodontal treatment that could bias outcomes; (5) Incomplete clinical or follow-up records, preventing reliable data analysis.

### Methods

All treatments were carried out by the same team of clinicians, each with more than 5 years of experience in periodontology and endodontics. Due to the particularity of the treatment methods, blinding was not implemented for the treating physicians in this study. However, all outcome indicator evaluations were completed by an independent researcher not involved in the treatment to reduce evaluation bias. All patients first underwent standardized root canal therapy. Preoperative periapical radiographs (Orthophos XG 3D, Dentsply Sirona, Germany) were obtained to assess root canal morphology and periapical status. Local anesthesia was administered using 4% articaine with epinephrine injection (containing epinephrine 1:100,000, Novocol Pharmaceutical of Canada Inc., Imported Drug Registration No.: HJ20220022, 1.7 mL: 68 mg/0.017 mg). The affected tooth was isolated with a rubber dam (Kerr Corporation, USA), and an access cavity was prepared. Root canals were explored with #10 and #15 K-files (VDW, Germany). Working length was established using an electronic apex locator (Root ZX, Morita, Japan) and verified radiographically. Root canal preparation was performed using ProTaper Gold nickel–titanium instruments (Dentsply Sirona, Germany) with a crown-down technique. During instrumentation, irrigation was alternated between 1% sodium hypochlorite (Shaanxi Hengrui Pharmaceutical Co., Ltd., Approval No.: H20193245) and 17% EDTA gel (Merck, Germany), followed by a final rinse with 0.9% sodium chloride solution (Shijiazhuang No. 4 Pharmaceutical Co., Ltd., Approval No.: H13023201, 500 mL: 4.5 g). After drying the canals, calcium hydroxide paste (Shanghai Second Medical Zhangjiang Biomaterials Co., Ltd., Approval No.: H20064332) was placed as an intracanal medicament, and the cavity was temporarily sealed. Patients were recalled after 1 week. If asymptomatic at follow-up, root canal obturation was completed using warm vertical compaction with thermoplasticized gutta-percha (System B heater and Obtura II injection system, SybronEndo, USA) and AH Plus sealer (Dentsply Sirona, Germany). Post-obturation radiographs were taken to confirm adequate filling before proceeding with periodontal flap surgery.

(1) Control group (periodontal flap surgery): Patients rinsed with 0.12% chlorhexidine solution (Letai Pharmaceutical Co., Ltd., Approval No.: H20064451, 300 mL) for 1 minute before surgery. Local infiltration anesthesia was administered using 4% articaine with epinephrine. Full-thickness mucoperiosteal flaps were elevated through internal bevel, sulcular, and vertical releasing incisions to fully expose the root surfaces and alveolar bone. Root planing was performed with Gracey curettes (Hu-Friedy, USA), and granulation tissue was thoroughly removed. The surgical site was irrigated with 0.9% sodium chloride solution, and the flap was repositioned and sutured using VICRYL absorbable sutures (Ethicon, Johnson & Johnson, USA; size VCP311H). A periodontal dressing (Zhengzhou Hongxin Medical Technology Co., Ltd.)was applied. Postoperative medications included oral amoxicillin–clavulanate (Guangzhou Baiyunshan Pharmaceutical Group Co., Ltd., Approval No.: H20041114, 0.457 g) and ibuprofen sustained-release capsules (Changzhou No. 4 Pharmaceutical Co., Ltd., Approval No.: H20093835, 0.3 g) for 3–5 days. Standard oral hygiene instructions were provided, and sutures were removed 10–14 days after surgery.
(2) Study group (dual-wavelength laser combined with periodontal flap surgery): Preoperative preparation, anesthesia, and flap elevation were identical to the control group. After flap reflection, additional dual-wavelength laser procedures were performed: (i) Nd: YAG laser irradiation of the root canal system: Root canal walls were irradiated using an Nd: YAG laser (SP Dynamis, Fotona, Germany; wavelength 1064 nm). The parameters were 1.5 W power and 15 Hz pulse frequency, delivered through a 300-μm fiber. Each canal was irradiated three times for 10 seconds per cycle with 20-second intervals, using a lifting motion. In addition, each canal irradiation operation strictly followed these parameter standards to ensure operational repeatability. (ii) Er: YAG laser treatment of periodontal tissues and root surfaces: An Er: YAG laser (SP Dynamis, Fotona, Germany; wavelength 2940 nm) was used to remove the pocket epithelium, debride inflammatory granulation tissue, and clean root surfaces. The settings were 100 mJ energy, 10 Hz frequency, and a 3:1 water–air ratio. Using the PS600 fiber handpiece, irradiation was applied in a non-contact circular motion at approximately 1–2 mm from the root surface until the surface was smooth and granulation tissue completely eliminated.

The postoperative medication regimens, oral hygiene instruction contents, and follow-up time points (follow-up on the 1st day, 7th day, 1st month, and 6th month after surgery) were completely the same for the two groups of patients.

### Outcome measures

(1) Pain assessment: Pain intensity was assessed using the Visual Analog Scale (VAS) (Huskisson, 1974) before periodontal flap surgery and at 1 and 7 days postoperatively. Patients were given a 0–10 VAS ruler (0 = no pain; 10 = worst imaginable pain) and asked to mark the number corresponding to their perceived pain level. All data were recorded by the same research assistant blinded to group allocation.
(2) Periodontal parameters: Periodontal measurements were obtained before root canal treatment and at 3 months postoperatively by a calibrated periodontist (intra-examiner correlation coefficient > 0.85) using a Williams periodontal probe (Hu-Friedy, USA). Six sites per affected tooth were examined (mesiobuccal, mid-buccal, distobuccal, mesiolingual, mid-lingual, distolingual). Recorded parameters included probing depth (PD; distance from the gingival margin to the pocket base), clinical attachment level (CAL; distance from the cementoenamel junction to the pocket base), GI (scored 0–3 using the Silness-Löe method (Loe and Silness, 1963), and plaque index (PLI, scored 0–3 using the Silness-Löe method (Loe and Silness, 1963).
(3) Inflammatory factors: Samples were collected before root canal treatment and at 3 months postoperatively. (i) Gingival crevicular fluid (GCF) collection: GCF was obtained after isolating and gently air-drying the sampling site. A standardized Periopaper strip (OraFlow, USA) was positioned at the base of the periodontal pocket for 30 seconds. Any strip contaminated by saliva or blood was discarded. The collected strips were immediately transferred to Eppendorf tubes and frozen at –80°C. Before measurement, each tube received 150 μL of PBS for elution. The eluates were then analyzed for interleukin-1β (IL-1β), interleukin-8 (IL-8), and matrix metalloproteinase-8 (MMP-8) using enzyme-linked immunosorbent assays (ELISAs). (ii) Serum collection: Five milliliters of fasting venous blood were attained in the morning and centrifuged at 3000 rpm for 10 minutes. The separated supernatant was aliquoted and stored at –80°C until testing. The same ELISA procedures were used to quantify IL-1β, IL-8, and MMP-8 in serum. All assay kits were supplied by Shanghai Enzyme-linked Biotechnology Co., Ltd., and all steps followed the manufacturer’s instructions.
(4) Bacterial infection and clearance rates: Subgingival plaque samples were obtained from the periodontal pockets of the affected teeth prior to treatment and again 3 months after root canal therapy. Sterile periodontal curettes (Hu-Friedy, USA) were utilized to collect the material, which was transferred into sterile Eppendorf tubes containing 1 mL PBS. Samples were immediately frozen at –80°C until processing (ultra-low temperature freezer, Haier, China; model DW-86L728). Genomic DNA was isolated using the cetyltrimethylammonium bromide (CTAB) protocol. DNA yield and purity were determined with a NanoDrop 2000 spectrophotometer (Thermo Fisher Scientific, USA). Quantification of three major periodontopathogens—*Porphyromonas gingivalis, Fusobacterium nucleatum*, and *Treponema denticola*—was performed via 16S rRNA real-time quantitative PCR (qPCR) using the CFX96 Touch platform (Bio-Rad, USA). Each 20-µL PCR reaction consisted of 10 µL SYBR Green Premix Pro Taq HS (Hunan Aikerui Biotechnology Co., Ltd., Cat. No. AG11701), 0.8 µL of each pathogen-specific primer (Shanghai Sangon Biotech), 2 µL DNA template, and 6.4 µL nuclease-free water. The cycling program included an initial denaturation at 95°C for 30 s, followed by 40 cycles of 95°C for 5 s and 60°C for 30 s, ending with melt-curve analysis. Ct values were processed using instrument software, and bacterial copy numbers were obtained from standard curves. A bacterial load > 1 × 10³ copies/µL was defined as positive. Bacterial infection rate was calculated as: (number of positive samples/total samples) × 100%. Bacterial clearance rate was calculated as: [(baseline positive samples – postoperative positive samples)/baseline positive samples] × 100%.
(5) Clinical efficacy: Clinical outcomes were evaluated 6 months following surgery according to the expert consensus criteria (Chen et al., 2024). Treatment was categorized as successful only when all of the following conditions were satisfied: (i) absence of patient-reported symptoms and restoration of normal masticatory function; (ii) no evidence of abscess formation or recurrence of sinus tracts; (iii) effective periodontal control, defined as PD ≤ 4 mm and radiographic improvement in alveolar bone height compared with preoperative images; (iv) resolution of pulpal and periapical inflammation, indicated by substantial reduction or disappearance of periapical radiolucency and reformation of the lamina dura. Failure to meet any criterion was classified as an unsuccessful outcome.
(6) Tooth status: At the 6-month postoperative follow-up, the presence of tooth mobility, periodontitis recurrence, and percussion pain was recorded for both groups.

### Statistical analysis

All statistical analyses were carried out using SPSS version 27.0. Categorical variables were summarized as counts and percentages, and intergroup differences were examined using either the chi-square test or Fisher’s exact test when appropriate. For continuous variables, distributional normality was evaluated with the Shapiro–Wilk test. Data that did not conform to a normal distribution were presented as median with interquartile range [M (P25, P75)], with between-group comparisons performed using the Mann–Whitney U test and within-group comparisons using the Wilcoxon signed-rank test. Variables meeting normality assumptions were expressed as mean ± standard deviation. Homogeneity of variance was verified through Levene’s test; equal variances warranted the use of the independent-samples t-test for between-group comparisons, whereas unequal variances required Welch’s t-test. Paired-samples t-tests were used for within-group analyses. For repeated measurements, repeated-measures ANOVA was applied when assumptions of normality and sphericity were met; otherwise, the Friedman test was employed. Multiple comparisons were adjusted using the Bonferroni procedure to control type I error inflation. A two-tailed significance threshold of α = 0.05 was adopted, and *P* < 0.05 was interpreted as statistically significant.

## Results

### VAS scores

Among the 106 enrolled participants (53 per group), 5 individuals in the study group (3 lost to follow-up; 2 withdrew) and 2 in the control group were excluded, leaving 99 patients for final analysis (48 in the study group; 51 in the control group).

Baseline VAS values were comparable between the two groups (*P* > 0.05). On postoperative day 1, pain levels increased in both groups; however, the study group reported lower VAS scores than the control group (*P* < 0.05). By day 7, VAS scores had markedly decreased from both baseline and day 1 levels in each group (*P* < 0.05), with no notable difference between the two groups at this time point (*P* > 0.05) (**Table 1**).

**Table 1:** Comparison of VAS scores between groups (points).

Time	Study group (n = 48)	Control group (n = 51)	Z	*P*
Pre-periodontal flap surgery	2.00 (2.00, 3.00)	2.00 (2.00, 3.00)	1.26	0.208
1 day post-surgery	4.00 (3.00, 4.00)^a^	4.00 (4.00, 5.00)^a^	4.581	< 0.001
7 days post-surgery	1.00 (1.00, 1.00)^ab^	1.00 (1.00, 1.00)^ab^	0.386	0.699

Compared with pre-surgery within the same group.

a *P* < 0.05; compared with 7 days post-surgery within the same group.

b *P* < 0.05.

### Periodontal parameters

Before root canal treatment, PD, CAL, GI, and PLI values did not differ markedly between groups (*P* > 0.05). At 3 months postoperatively, all four parameters showed remarkable improvement in both groups, and the reductions observed in the study group were greater than those in the control group (*P* < 0.05) (**Table 2**).

**Table 2:** Comparison of periodontal parameters between groups.

Time	Study group (n = 48)	Control group (n = 51)	t/Z	*P*
Before root canal treatment				
PD (mm)	6.26 ± 0.76	6.34 ± 0.85	0.534	0.595
CAL (mm)	5.80 ± 0.72	5.77 ± 0.76	0.225	0.822
GI (points)	2.45 (2.20, 2.70)	2.50 (2.20, 2.70)	0.636	0.525
PLI (points)	2.35 (2.05, 2.50)	2.30 (2.00, 2.50)	0.292	0.77
3 months postoperatively				
PD (mm)	2.86 ± 0.42^a^	3.17 ± 0.53^a^	3.126	0.002
CAL (mm)	3.73 ± 0.56^a^	4.05 ± 0.62^a^	2.705	0.008
GI (points)	0.40 (0.20, 0.50)^a^	0.60 (0.35, 0.70)^a^	3.206	0.001
PLI (points)	0.30 (0.10, 0.35)^a^	0.40 (0.20, 0.50)^a^	3.592	< 0.001

Compared with pre-root canal treatment within the same group.

a *P* < 0.05.

### GCF inflammatory factors

Levels of IL-1β, IL-6, and MMP-8 in GCF did not differ noticeably between the two groups at baseline (*P* > 0.05). At 3 months postoperatively, each marker declined in both groups, with the study group exhibiting lower concentrations than the control group (*P* < 0.05) (**Table 3**).

**Table 3:** Comparison of gingival crevicular fluid inflammatory factors between groups.

Time	Study group (n = 48)	Control group (n = 51)	t	*P*
Before root canal treatment				
IL-1β (pg/mL)	376.87 ± 52.03	381.54 ± 54.08	0.438	0.663
IL-6 (pg/mL)	86.01 ± 11.48	85.58 ± 12.52	0.179	0.859
MMP-8 (ng/mL)	1256.34 ± 162.20	1238.91 ± 160.90	0.537	0.593
3 months postoperatively				
IL-1β (pg/mL)	117.62 ± 21.38^a^	132.63 ± 28.74^a^	2.959	0.004
IL-6 (pg/mL)	24.75 ± 6.74^a^	32.45 ± 7.83^a^	5.236	< 0.001
MMP-8 (ng/mL)	285.71 ± 60.41^a^	352.88 ± 72.71^a^	4.983	< 0.001

Compared with pre-root canal treatment within the same group.

a *P* < 0.05.

### Serum inflammatory factors

Similar trends were observed in serum cytokine levels. Baseline serum IL-1β, IL-6, and MMP-8 concentrations were comparable between groups (*P* > 0.05). All three biomarkers decreased significantly after 3 months, and the study group again demonstrated lower levels than the control group (*P* < 0.05) (**Table 4**).

**Table 4:** Comparison of serum inflammatory factors between groups.

Time	Study group (n = 48)	Control group (n = 51)	t	*P*
Before root canal treatment				
IL-1β (pg/mL)	8.45 ± 2.13	8.27 ± 2.27	0.424	0.673
IL-6 (pg/mL)	15.58 ± 3.13	15.25 ± 3.04	0.533	0.595
MMP-8 (ng/mL)	280.26 ± 40.54	273.75 ± 39.82	0.806	0.422
3 months postoperatively				
IL-1β (pg/mL)	3.86 ± 1.43^a^	4.46 ± 1.50^a^	2.022	0.046
IL-6 (pg/mL)	6.38 ± 1.63^a^	7.19 ± 1.76^a^	2.377	0.019
MMP-8 (ng/mL)	72.27 ± 12.48^a^	79.87 ± 11.85^a^	3.111	0.002

Compared with pre-root canal treatment within the same group.

a *P* < 0.05.

### Bacterial infection and clearance rates

The incidence of bacterial infection prior to treatment did not differ remarkably between groups (*P* > 0.05). At 3 months postoperatively, infection rates had declined while bacterial clearance rates had increased in both groups. Notably, the study group showed a lower infection rate and a higher clearance rate relative to the control group (*P* < 0.05) (**Table 5**).

**Table 5:** Comparison of bacterial infection and clearance rates between groups [n (%)].

Indicator	Study group (n = 48)	Control group (n = 51)	χ^2^	*P*
Bacterial infection rate				
Before root canal treatment	45 (93.75)	48 (94.12)	*	1.000
3 months postoperatively	3 (6.25)^a^	12 (23.53)^a^	5.743	0.017
Bacterial clearance rate	42 (93.33)	36 (75.00)	5.771	0.016

*Fisher’s exact test; Compared with pre-root canal treatment within the same group.

a *P* < 0.05.

### Clinical efficacy and tooth status

At 6 months postoperatively, the study group had a higher overall treatment success rate (95.83% vs. 82.35%) and lower rates of tooth mobility (2.08% vs. 15.69%), periodontitis recurrence (4.17% vs. 17.65%), and percussion pain (6.25% vs. 19.61%) in contrast to the control group (*P* < 0.05) (**Table 6**).

**Table 6:** Comparison of clinical efficacy and tooth status between groups [n (%)].

Complication	Study group (n = 48)	Control group (n = 51)	χ^2^	*P*
Treatment success rate	46 (95.83)^a^	42 (82.35)	4.55	0.033
Tooth mobility rate	1 (2.08)^a^	8 (15.69)	*	0.032
Periodontitis recurrence rate	2 (4.17)^a^	9 (17.65)	4.55	0.033
Percussion pain rate	3 (6.25)^a^	10 (19.61)	3.868	0.049

*Fisher’s exact test. Compared with the control group.

a *P* < 0.05.

## Discussion

PEL represents a complex pathological condition in which microorganisms invade both periodontal and pulpal tissues through interconnected anatomical pathways (Zeng et al., 2024). Factors such as periodontal destruction, pulpal degeneration, root resorption, trauma, and perforation further facilitate microbial penetration and accelerate PEL progression (Wong et al., 2024). Owing to their multifocal infection routes and heterogeneous presentations, PEL remain challenging to diagnose and treat and are an important cause of tooth loss (Yan et al., 2021). The results of this study indicate that for patients with combined periodontal-endodontic lesions, dual-wavelength laser-assisted periodontal flap surgery demonstrates superior effects compared to periodontal flap surgery alone in terms of postoperative analgesia, improvement of periodontal clinical indicators, regulation of inflammatory factors, microbial clearance, and long-term success rate.

Patients receiving the laser-assisted protocol experienced lower postoperative VAS scores, consistent with meta-analytic evidence showing that adjunctive photobiomodulation reduces intra- and postoperative pain during periodontal procedures (Mikami et al., 2020). Similar to the findings of this study, Roy et al. employed a 940 nm diode laser-assisted flap surgery and found that the VAS scores in the laser group are lower than those in the control group on the 1st and 3rd days after surgery, but the difference in pain disappeared over a longer period (Roy et al., 2022). Additionally, Vineet et al. used a 445 nm laser-assisted Kirkland flap surgery and also reported lower VAS scores at the laser test sites compared to the control sites (V et al., 2024). The early analgesic mechanism of lasers is mainly related to the photobiomodulation effect, which inhibits peripheral nociceptors and reduces the production of pain-inducing mediators such as bradykinin and prostaglandin E2. Moreover, the thermal effect of lasers can form a transient “bio-sealing layer” (Tomazoni et al., 2021; Roy et al., 2022; Uta et al., 2023). Since the peak of acute postoperative pain occurs within 24–72 hours, the inter-group differences are mainly concentrated in this time window.

The dual-wavelength protocol also produced superior improvements in PD, CAL, GI, and PLI at three months, illustrating enhanced periodontal healing. One clinical trial demonstrates that diode-laser–assisted flap surgery yields greater reductions in pocket depth and increased CAL gain over 6 months compared with flap surgery alone (Agarwal et al., 2021). Furthermore, Vineet et al.’s study demonstrated that the 445 nm laser-assisted group has superior PD reduction and CAL gain compared to the control group at 1, 3, and 6 months after surgery (V et al., 2024). Roy et al.’s study showed significant improvements in PD and CAL within all groups at six months after surgery, but the inter-group differences between the laser and control groups do not reach statistical significance (Roy et al., 2022). These inconsistencies may be related to factors such as laser wavelength, output mode, and lesion type.

This study also showed that the levels of IL-1β, IL-6, and MMP-8 in GCF were lower in the study group than in the control group at three months after surgery. It has been reported that photobiomodulation therapy (PBMT)-driven enhancement of cytochrome-c oxidase activity, ATP synthesis, and nitric oxide and ROS regulation, which collectively suppress early inflammatory mediator release (Dompe et al., 2020). *In vitro* data showing that PBMT stimulates periodontal ligament stem cell proliferation and reduces IL-1β and IL-6 expression (Wang et al., 2022) further support the improved early wound comfort observed in this study. Moreover, diode laser treatment significantly decreases GCF MMP-8 and improves periodontal stability at 3–6 months (Sopi et al., 2023). Because active MMP-8 correlates strongly with disease severity and ongoing periodontal destruction (Sorsa et al., 2020), its marked reduction in this study indicates more effective suppression of destructive inflammatory pathways. PBMT’s ability to attenuate oxidative stress and inflammatory cytokine transcription (Dompe et al., 2020) offers further mechanistic support for these observations.

The study group demonstrated higher bacterial elimination and lower residual infection at three months. Although diode lasers used solely with nonsurgical scaling and root planing sometimes show limited microbial benefits (Seyed-Monir et al., 2023), laser irradiation has been shown to disrupt biofilm structure and increase bacterial susceptibility to mechanical removal (Wang et al., 2022). These effects are especially relevant in PEL, where deep pockets, root concavities, and periodontal–endodontic communication pathways limit the efficacy of traditional debridement. This combination of improved mechanical access via flap surgery and photonic biofilm disruption provides a strong rationale for the superior microbial clearance observed. These results are consistent with evidence regarding the antibacterial efficacy of laser-assisted therapy. Diode et al.’s 976 nm/650 nm multi-stage laser protocol for refractory combined periodontal-endodontic lesions also reported that laser assistance can achieve deep biofilm clearance and improvement of clinical parameters (Munteanu et al., 2025).

Importantly, the therapeutic benefits of dual-wavelength laser–assisted surgery were sustained at six months, reflected in higher overall treatment success, reduced tooth mobility, fewer recurrence-related symptoms, and improved functional stability. These results align with prior evidence showing more stable CAL preservation and bone-level improvement following laser-assisted periodontal therapy during medium-term follow-up (Agarwal et al., 2021; Sopi et al., 2023). In addition, PBMT has been shown to promote fibroblast and periodontal ligament stem cell proliferation, enhance collagen remodeling, and support soft-tissue repair (Wang et al., 2022), which may help explain the greater periodontal stability observed in this study. Existing evidence supports that all 12 refractory cases showed measurable improvements after the multi-stage laser protocol, with the median probing depth decreasing from 7.6 mm to 6.0 mm (Munteanu et al., 2025). From a clinical perspective, the significant reductions in tooth mobility rate and percussion pain rate are unique evaluation dimensions for the treatment of combined periodontal-endodontic lesions. The results of this study suggest that laser-assisted therapy has practical value in improving the functional prognosis of affected teeth.

This study has the following limitations: It did not conduct stratified analysis by tooth type (anterior teeth, premolars, molars). Different tooth types have differences in anatomical structure, number of root canals, degree of furcation involvement, surgical approach, and accessibility of the laser optical fiber, which may introduce heterogeneity in the evaluation of treatment effects. Additionally, the single-center design, relatively limited sample size, and six-month follow-up period are insufficient to assess long-term periodontal stability and tooth survival rate.

Taken together, this study demonstrates that dual-wavelength laser adjunctive therapy enhances the clinical efficacy of periodontal flap surgery in PEL by improving periodontal healing, reducing inflammatory burden, and promoting bacterial clearance, ultimately supporting better medium-term clinical stability than flap surgery alone. Clinically, these findings suggest that integrating dual-wavelength laser technology into conventional surgical protocols may provide a more predictable therapeutic pathway for managing complex periodontal–endodontic lesions, thereby improving long-term functional and oral health outcomes for affected patients. Subsequent studies should expand the sample size and adopt a stratified randomized design based on anterior teeth, premolars, and molars to more accurately evaluate the modifying effects of different tooth types on treatment effects and avoid heterogeneity interference. Meanwhile, the follow-up period could be extended to over one year, incorporating radiographic bone fill evaluation and patient-reported outcome measures. Furthermore, the molecular mechanisms by which dual-wavelength lasers regulate the host inflammatory-repair transition should be further explored to provide higher-level evidence-based support for their clinical standardized application.

## Data Availability

The raw data supporting the conclusions of this article will be made available by the authors, without undue reservation.
